# Association of Chronological Age and Comorbidity Burden with Outcomes After Proton Beam Therapy for Hepatocellular Carcinoma

**DOI:** 10.3390/cancers18183005

**Published:** 2026-09-16

**Authors:** Haruka Shirataki, Masashi Mizumoto, Takashi Iizumi, Takashi Saito, Haruko Numajiri, Masahiko Harada, Takuya Sawada, Masaaki Goto, Taisuke Sumiya, Takeshi Fujisawa, Keiichiro Baba, Masatoshi Nakamura, Kei Nakai, Hideyuki Sakurai

**Affiliations:** Department of Radiation Oncology, University of Tsukuba, Tsukuba 305-8575, Ibaraki, Japan

**Keywords:** proton beam therapy, hepatocellular carcinoma, Charlson Comorbidity Index, comorbidity, radiotherapy

## Abstract

Background/Objectives: The prognostic relevance of chronological age versus comorbidity burden in patients with hepatocellular carcinoma (HCC) undergoing proton beam therapy (PBT) is unclear. This study evaluated whether comorbidity burden provides prognostic information beyond age. Methods: We retrospectively analyzed 269 patients with HCC treated with PBT between December 2009 and March 2015. Patients were classified as elderly (≥75 years, *n* = 105) or non-elderly (<75 years, *n* = 164). Overall survival (OS), local control (LC), progression-free survival (PFS), and treatment-related adverse events were assessed. Comorbidity burden was evaluated using the Charlson Comorbidity Index (CCI). Multivariable Cox regression analyses were performed for OS and PFS. Results: The 3-year OS rates were 65.9% in the elderly group and 56.9% in the non-elderly group (*p* = 0.20), while the corresponding LC rates were 86.7% and 90.5% (*p* = 0.24) and PFS rates were 30.3% and 29.9% (*p* = 0.81), respectively. In multivariable analyses, age ≥ 75 years was not significantly associated with OS (HR, 1.25; 95% CI, 0.89–1.74; *p* = 0.198) or PFS (HR, 1.15; 95% CI, 0.86–1.54; *p* = 0.331). In contrast, CCI ≥ 3, compared with CCI 0, was independently associated with poorer OS (HR, 3.31; 95% CI, 1.54–7.15; *p* = 0.002), while its association with PFS did not reach statistical significance (HR, 1.87; 95% CI, 0.98–3.58; *p* = 0.057). No significant age × CCI interaction was observed for OS or PFS. Conclusions: Chronological age was not independently associated with OS or PFS in patients with HCC treated with PBT, whereas a high comorbidity burden was independently associated with poorer OS. These findings suggest that comorbidity burden should be considered in addition to chronological age when estimating prognosis after PBT.

## Simple Summary

Hepatocellular carcinoma often occurs in older adults, but chronological age alone may not accurately reflect a patient’s overall health or prognosis. This study evaluated whether comorbidities, rather than age itself, were associated with outcomes after proton beam therapy for hepatocellular carcinoma. We compared survival, tumor control, disease progression, treatment-related adverse events, and causes of death between older and younger patients, and also examined the impact of comorbidity burden. Severe treatment-related toxicities were uncommon in both age groups, whereas a greater comorbidity burden was associated with poorer overall survival. Although subgroup analyses showed different patterns between age groups, formal interaction testing did not confirm that the prognostic effect of comorbidity differed according to age. These findings suggest that comorbidity burden should be considered in addition to chronological age when estimating prognosis after proton beam therapy.

## 1. Introduction

Hepatocellular carcinoma (HCC) remains a leading cause of cancer-related mortality worldwide and is an important clinical concern in aging populations such as Japan. Recent nationwide data have demonstrated substantial geographic variation in the incidence of HCC in Japan, while treatment patterns remain relatively consistent across regions, suggesting that HCC is managed within a broadly standardized national treatment framework despite differences in regional disease burden [1]. Potentially curative treatments for HCC include surgical resection, radiofrequency ablation, and liver transplantation. However, these approaches are not feasible for a substantial proportion of patients because of impaired liver function, unfavorable tumor size or location, vascular invasion, comorbidities, or other factors that limit surgical or invasive local treatment, underscoring the need for effective alternative locoregional therapies [2,3,4]. Radiotherapy has increasingly become an important non-invasive local treatment option for patients with HCC who are not suitable candidates for surgery or other invasive locoregional therapies. In particular, advances in image guidance, motion management, and highly conformal dose delivery have enabled the use of stereotactic body radiotherapy (SBRT), allowing high doses to be delivered to the tumor over a limited number of fractions while reducing irradiation of the surrounding liver. Recent systematic reviews and meta-analyses have further supported the clinical role of SBRT in early-stage HCC and have also evaluated its outcomes in comparison with other established locoregional treatments such as transarterial chemoembolization [5,6]. In parallel, particle therapies, including proton beam therapy (PBT) and carbon-ion radiotherapy, have been investigated as highly conformal treatment approaches for HCC, with increasing clinical evidence summarized in recent reviews and meta-analyses [7,8]. Among these modalities, PBT has particular dosimetric advantages because of its characteristic depth-dose distribution, allowing high radiation doses to be delivered to the tumor while reducing unnecessary irradiation of the surrounding normal liver. This property may be especially relevant in patients with underlying chronic liver disease, in whom preservation of functional liver parenchyma is an important consideration during treatment planning. Previous clinical studies have demonstrated high local control rates and favorable survival outcomes with PBT, supporting its use as a definitive local treatment option for selected patients with HCC [9,10]. Taken together, these advances have expanded the role of radiotherapy from a predominantly palliative modality toward a potentially curative local treatment strategy in appropriately selected patients.

Several prognostic factors after PBT for HCC have been reported, including tumor size, liver function, and portal vein tumor thrombosis [11,12,13]. However, the prognostic significance of chronological age remains less clearly defined. This issue is particularly relevant because HCC frequently occurs in older adults, who often have multiple comorbidities and varying degrees of physiological reserve in addition to underlying chronic liver disease. Although elderly patients may be considered more vulnerable to cancer treatment, chronological age itself does not necessarily reflect an individual patient’s functional status, comorbidity burden, or ability to tolerate treatment. Indeed, vulnerability in older adults is a multidimensional concept that cannot be adequately characterized by age alone [14]. Therefore, assessment of factors beyond chronological age may be important when estimating prognosis and considering treatment strategies for older patients with HCC.

Comorbidity burden and other measures of physiological vulnerability may provide clinically relevant prognostic information in addition to chronological age. In older adults with cancer, increasing attention has therefore been directed toward frailty assessment, geriatric assessment, and other multidimensional approaches that evaluate functional and physiological status rather than relying solely on chronological age [15,16]. Among the available measures of comorbidity, the Charlson Comorbidity Index (CCI) is a widely used tool for quantifying comorbidity burden and estimating mortality risk. The potential value of comorbidity assessment has also been investigated specifically in patients with HCC, and modified CCI-based approaches have been proposed as part of multiparametric prognostic assessment [17,18]. However, the prognostic significance of comorbidity burden in relation to chronological age remains insufficiently characterized in patients with HCC undergoing PBT. In particular, it remains unclear whether chronological age itself is associated with treatment outcomes after PBT or whether comorbidity burden provides additional prognostic information after accounting for other established clinical and tumor-related factors. Therefore, in this study, we compared survival outcomes, local tumor control, and treatment-related adverse events between elderly (≥75 years) and non-elderly patients with HCC treated with PBT and further investigated the prognostic significance of comorbidity burden assessed using the CCI.

## 2. Materials and Methods

### 2.1. Study Design and Patient Population

A retrospective cohort study was performed to evaluate the safety and efficacy of PBT for HCC in patients treated at the University of Tsukuba Hospital between December 2009 and March 2015. PBT candidacy was determined based on overall clinical assessment. In general, patients with distant metastases, Child-Pugh class C liver function, inability to maintain the required treatment position, or other active lesions expected to determine prognosis were generally considered unsuitable candidates for PBT. In addition, PBT was generally considered when all intrahepatic lesions present at the time of treatment were planned to be treated with PBT, or when remaining lesions were scheduled to receive other local treatments such as radiofrequency ablation or transarterial chemoembolization. Performance status alone was not an absolute exclusion criterion; patients with poor performance status could still receive PBT when the disease was localized and local control was considered likely to provide clinical benefit.

Patients were classified as elderly (≥75 years) or non-elderly (<75 years) according to age at PBT initiation. The study was approved by the Institutional Review Board (H29-151) of the University of Tsukuba Hospital. Given the retrospective design, the requirement for study-specific informed consent was waived; however, all patients had provided general consent for treatment and use of their clinical data for research.

### 2.2. Treatment Protocol

All patients underwent PBT according to institutional protocols that have been described previously. Briefly, treatment schedules were selected according to tumor location: 66 Gy (RBE) in 10 fractions for peripheral lesions, 72.6 Gy (RBE) in 22 fractions for hilar lesions, and 74 Gy (RBE) in 37 fractions for lesions adjacent to the gastrointestinal tract. Alternative planned regimens of 60 Gy (RBE) in 15 fractions and 70 Gy (RBE) in 35 fractions were used in a small number of patients. The prescribed dose and fractionation schedules were determined based on tumor location, size, and proximity to critical structures, in accordance with Japanese national guidelines and clinical judgment [2].

### 2.3. Data Collection and Outcome Measures

Clinical data were collected from medical records, including age, sex, performance status (PS), liver function (Child-Pugh score, platelet count, AST, and ALT), tumor characteristics (tumor size, portal vein tumor thrombosis status, AFP, and DCP), and comorbidities. Comorbidity burden was assessed using the CCI, a validated tool for estimating mortality risk. Comorbidities present at the time of treatment were scored. The primary endpoints were overall survival (OS), local control (LC), and progression-free survival (PFS). OS was defined as the time from initiation of PBT to death from any cause, LC as the absence of tumor progression within the irradiated field, and PFS as the time from initiation of PBT to disease progression or death from any cause. Tumor progression and local recurrence were assessed by comparison with previous follow-up imaging. Progressive disease was generally defined as a clear increase in tumor size. In cases without obvious tumor enlargement, local recurrence was determined based on a comprehensive clinical assessment, including enlargement of the contrast-enhancing component, elevation of tumor markers, and the absence of other evaluable sites of recurrence such as out-of-field or distant disease. These assessments were based on routine clinical practice rather than a prospectively predefined uniform radiologic response criterion.

Treatment-related adverse events were evaluated using the Common Terminology Criteria for Adverse Events (CTCAE) version 4.0 and were analyzed as a secondary endpoint [19].

### 2.4. Statistical Analysis

Comparisons between the elderly and non-elderly groups were performed for all endpoints. Survival outcomes, including OS, LC, and PFS, were estimated using the Kaplan–Meier method, with differences between groups assessed by the log-rank test. Baseline characteristics were compared by chi-square test or Fisher’s exact test, as appropriate. The prognostic impact of comorbidity burden was evaluated in subgroup analyses performed according to CCI categories (CCI 0, 1–2, and ≥3). For the exploratory analysis of causes of death, CCI was dichotomized as 0–3 versus 4–6 because of the small number of events in individual CCI categories. Multivariable Cox proportional hazards regression analyses were performed for OS and PFS to evaluate the independent associations of age and CCI after adjustment for clinically relevant covariates, including PS, Child-Pugh score, tumor size, PVTT, AFP, sex, etiology, and platelet count. Age was entered as a binary variable (≥75 vs. <75 years), and CCI was categorized as 0, 1–2, and ≥3. Because the 75-year cutoff was pragmatic rather than based on a biologically validated threshold, a sensitivity analysis was also performed with age treated as a continuous variable. Formal age group × CCI interaction terms were tested for both OS and PFS. Hazard ratios (HRs) with 95% confidence intervals (CIs) were calculated. Age was not included in the CCI calculation; the original CCI rather than the age-adjusted CCI was used. Because the study population consisted exclusively of patients with HCC, HCC itself was excluded from the CCI calculation to avoid incorporation of the index disease into the comorbidity assessment. Other liver disease components included in the original CCI were retained in the calculation. A two-sided *p*-value < 0.05 was considered significant. All statistical analyses were performed using R, version 4.3.1 (R Foundation for Statistical Computing Vienna, Austria).

## 3. Results

### 3.1. Patient Characteristics

A total of 269 patients with HCC underwent PBT in the study period, comprising 105 elderly (≥75 years) and 164 non-elderly (<75 years) patients. The median follow-up period for survivors was 37 months (range, 1–131 months). Baseline characteristics are summarized in Table 1. There were significant differences between the groups in sex distribution, etiology, platelet count, PVTT, and PS, whereas tumor size and Child-Pugh score were similar. Most patients in both groups had Child-Pugh class A liver function and good PS (ECOG 0–2). CCI was similar in the two groups (*p* = 0.638). The distribution of PBT dose-fractionation regimens also did not differ significantly between the two groups (*p* = 0.422). No unplanned dose reductions, fractionation changes, or treatment discontinuations occurred after initiation of PBT, and all patients completed the planned treatment course.

### 3.2. Overall Survival (OS)

**Figure 1:**
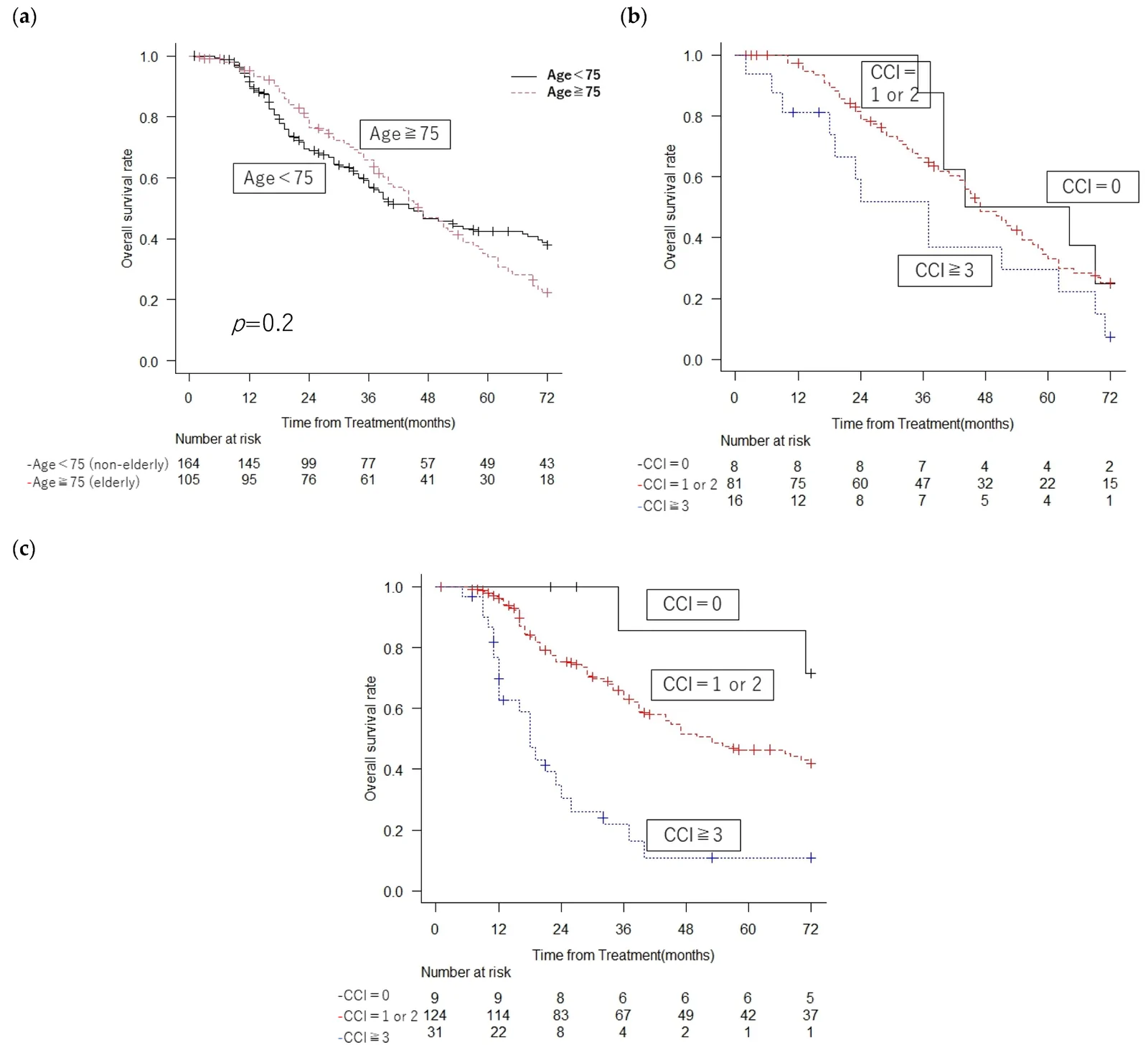
Overall survival (OS) according to age and comorbidity burden. (**a**) Kaplan–Meier curves for OS in elderly (≥75 years) and non-elderly (<75 years) patients. (**b**) OS stratified by Charlson Comorbidity Index (CCI) in elderly patients. (**c**) OS stratified by CCI in non-elderly patients.

OS did not differ significantly in the elderly and non-elderly groups (log-rank *p* = 0.20). The 1-, 2-, and 3-year OS rates were 95.1% (95% CI, 88.6–97.9%), 76.6% (66.9–83.8%), and 65.9% (55.4–74.4%) in the elderly group, and 89.9% (84.0–93.7%), 68.9% (60.8–75.6%), and 56.9% (48.4–64.6%) in the non-elderly group, respectively (Figure 1a). Stratified by CCI, OS showed no significant difference among CCI subgroups in the elderly group (*p* = 0.17), but CCI significantly influenced OS in the non-elderly group (*p* < 0.001), with worse survival in patients with a higher CCI (Figure 1b,c).

### 3.3. Local Control (LC)

**Figure 2:**
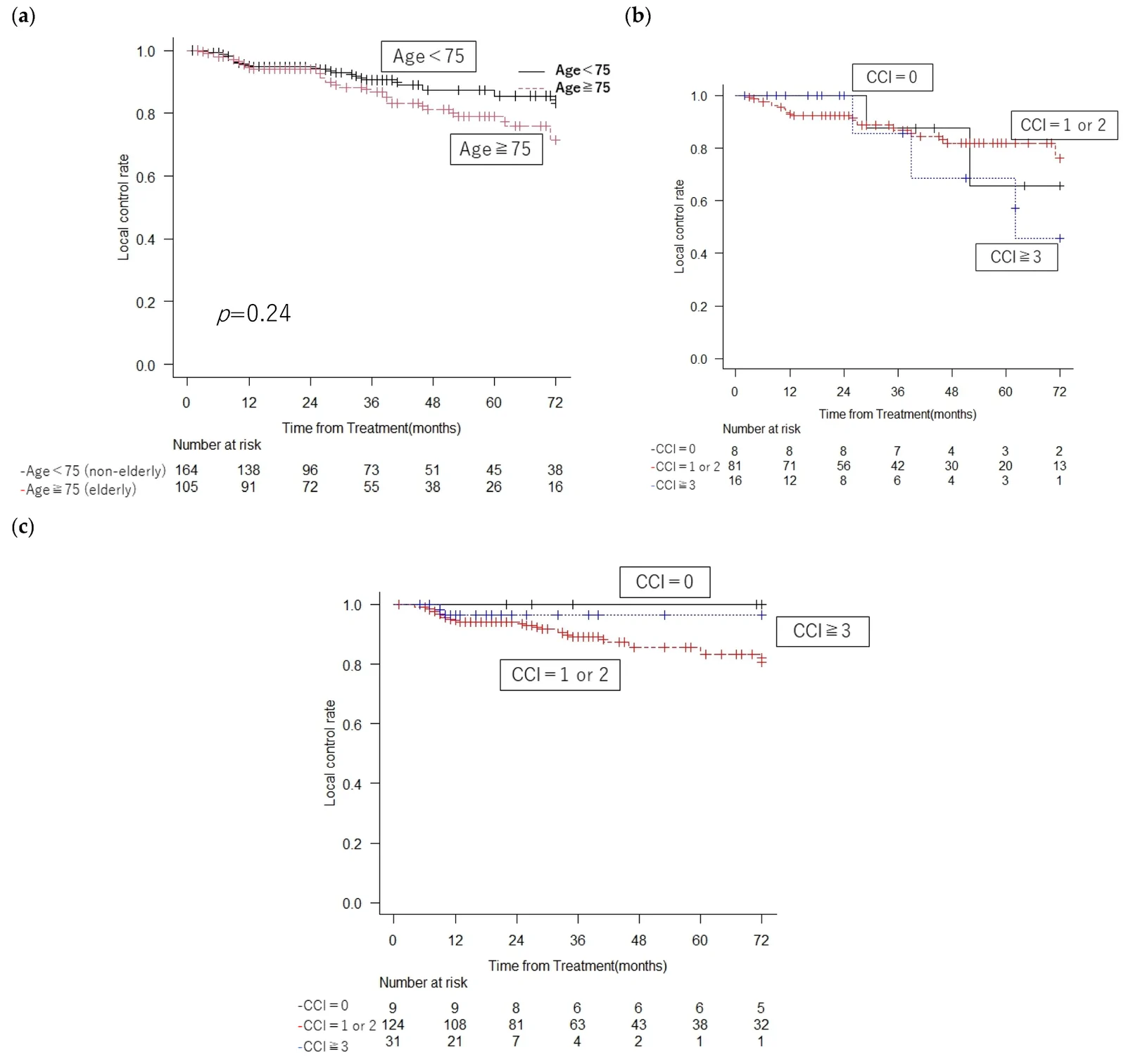
Local control (LC) according to age and comorbidity burden. (**a**) Kaplan–Meier curves for LC in elderly and non-elderly patients. (**b**) LC stratified by Charlson Comorbidity Index (CCI) in elderly patients. (**c**) LC stratified by CCI in non-elderly patients.

The 1-, 2-, and 3-year LC rates were 93.9% (95% CI, 87.0–97.2%), 93.9% (87.0–97.2%), and 86.7% (77.0–92.5%) in the elderly group, and 94.9% (90.0–97.4%), 94.9% (90.0–97.4%), and 90.5% (83.6–94.6%) in the non-elderly group, respectively (Figure 2a), with no significant difference in LC between the two groups (log-rank *p* = 0.24). Stratification by CCI showed no significant differences in LC among CCI subgroups (Figure 2b,c) in the elderly (*p* = 0.685) and non-elderly (*p* = 0.687) groups.

### 3.4. Progression-Free Survival (PFS)

**Figure 3:**
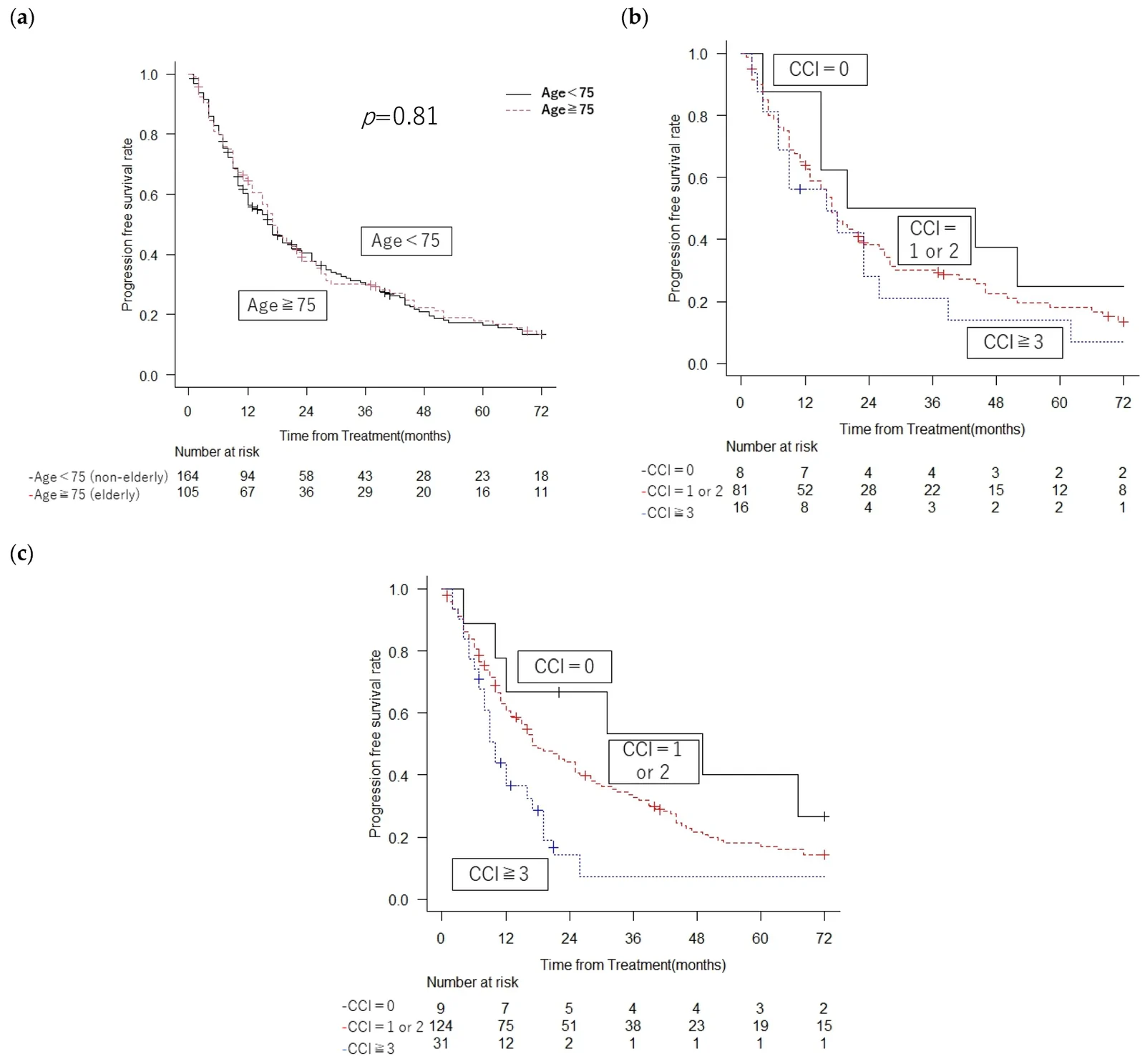
Progression-free survival (PFS) according to age and comorbidity burden. (**a**) Kaplan–Meier curves for PFS in elderly and non-elderly patients. (**b**) PFS stratified by Charlson Comorbidity Index (CCI) in elderly patients. (**c**) PFS stratified by CCI in non-elderly patients.

The 1-, 2-, and 3-year PFS rates were 63.5% (95% CI, 53.5–71.9%), 37.6% (28.3–46.9%), and 30.3% (21.6–39.4%) in the elderly group, and 56.5% (48.5–63.8%), 40.5% (32.8–48.1%), and 29.9% (22.8–37.4%) in the non-elderly group, respectively (Figure 3a). There was no significant difference in PFS between the two groups (log-rank *p* = 0.81). Stratified by CCI, there was no significant difference in PFS among CCI subgroups (Figure 3b,c) in the elderly group (*p* = 0.351), but there were significant differences in the non-elderly group (*p* = 0.0075).

### 3.5. Multivariable Analysis

Multivariable Cox proportional hazards regression analyses for OS and PFS are summarized in Table 2. For OS, age ≥ 75 years was not significantly associated with mortality (HR, 1.25; 95% CI, 0.89–1.74; *p* = 0.198), whereas CCI ≥ 3, compared with CCI 0, was independently associated with poorer OS (HR, 3.31; 95% CI, 1.54–7.15; *p* = 0.002). Child-Pugh score, tumor size, and sex were also significantly associated with OS. For PFS, age ≥ 75 years was not significantly associated with outcome (HR, 1.15; 95% CI, 0.86–1.54; *p* = 0.331). CCI ≥ 3 showed a trend toward poorer PFS but did not reach statistical significance (HR, 1.87; 95% CI, 0.98–3.58; *p* = 0.057). Child-Pugh score, tumor size, PVTT, and sex were significantly associated with PFS.

In a sensitivity analysis treating age as a continuous variable, age was not significantly associated with PFS (HR, 1.01; 95% CI, 0.99–1.03; *p* = 0.189) and showed only a borderline association with OS (HR, 1.02; 95% CI, 1.00–1.04; *p* = 0.052). The association between CCI ≥ 3 and poorer OS remained significant in this analysis (HR, 3.29; 95% CI, 1.53–7.11; *p* = 0.002). Formal interaction testing showed no statistically significant age group × CCI interaction for either OS (*p* = 0.094) or PFS (*p* = 0.903).

### 3.6. Adverse Events

Treatment-related adverse events are summarized in Table 3. No acute Grade ≥ 3 adverse events were observed in either age group. The most common acute adverse event was Grade 1–2 dermatitis, occurring in 45 elderly patients (42.9%) and 44 non-elderly patients (26.8%). Late Grade ≥ 3 adverse events were uncommon and occurred only in the non-elderly group, including one case each of gastrointestinal bleeding and dermatitis.

### 3.7. Causes of Death

**Figure 4:**
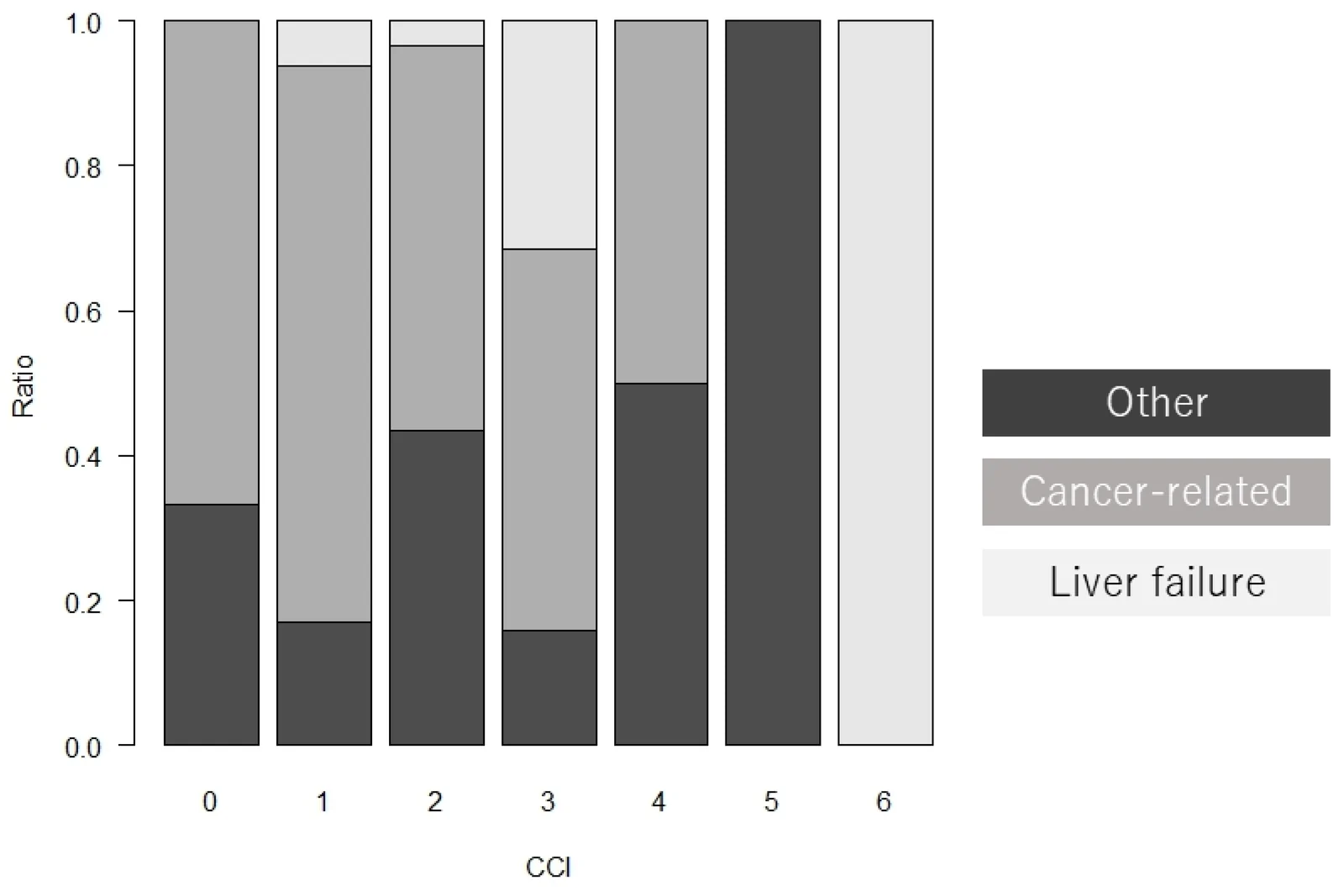
Distribution of causes of death according to Charlson Comorbidity Index (CCI).

Causes of death according to CCI are shown in Figure 4. Among patients with CCI 0–3, death was due to cancer (66.7%), other causes (24.2%), and liver failure (9.2%). In contrast, for patients with a higher comorbidity burden (CCI 4–6), these rates were 33.3%, 55.6% and 11.1%, respectively. The distribution of causes of death did not differ significantly between the CCI groups (Fisher’s exact test, *p* = 0.0934).

## 4. Discussion

This study showed that survival outcomes after PBT for HCC were similar between elderly and non-elderly patients. In multivariable analyses, chronological age was not independently associated with OS or PFS, whereas a high comorbidity burden (CCI ≥ 3) was independently associated with poorer OS; its association with PFS did not reach statistical significance. Although subgroup analyses showed different patterns across age groups, formal interaction testing did not demonstrate a statistically significant age × CCI interaction for either OS or PFS. Therefore, the subgroup findings should not be interpreted as evidence that the prognostic effect of CCI differs according to age.

One possible explanation for the observed pattern is the influence of competing risks of death. In elderly patients with cancer, non-cancer-related mortality becomes increasingly important and may contribute substantially to overall survival independently of cancer control. Previous studies have highlighted the importance of non-cancer mortality in older patients after potentially curative cancer treatment [20]. More broadly, both chronological age and comorbidity have been recognized as important determinants of outcomes in elderly patients with cancer, reflecting the contribution of competing health risks in addition to the malignancy itself [21]. In the present study, the proportion of non-cancer-related deaths was numerically higher among patients with a greater comorbidity burden; however, the overall distribution of causes of death did not differ significantly between CCI groups (Fisher’s exact test, *p* = 0.0934). Thus, competing mortality may partly contribute to the observed pattern, although this interpretation remains hypothesis-generating. Because the formal interaction between age group and CCI was not statistically significant, these findings should not be interpreted as evidence that the prognostic effect of comorbidity differs definitively according to age. Rather, they suggest that the relationship among age, comorbidity burden, cancer-related mortality, and competing causes of death is complex and warrants cautious interpretation.

Notably, LC did not differ significantly according to age or comorbidity burden in the present cohort. Previous prospective studies have also demonstrated favorable local control after high-dose hypofractionated PBT for HCC [13]. However, tumor control in HCC is influenced not only by treatment-related factors but also by intrinsic tumor biology. Peng et al. reported that PSMD12 was overexpressed in HCC and that higher expression was associated with poorer prognosis; mechanistically, PSMD12 promoted HCC cell proliferation and migration by stabilizing CDK1 through deubiquitination [22]. These findings illustrate that biological characteristics of the tumor may contribute to disease progression independently of patient background factors such as age or comorbidity burden.

Previous studies have reported favorable local control and toxicity outcomes with PBT for HCC [9,11,23,24,25]. However, the present study was not designed to compare PBT with other treatment modalities or to evaluate a dose–response relationship. Our findings are consistent with emerging evidence supporting the prognostic significance of comorbidity burden in patients with HCC. In a Swedish nationwide registry study of 980 patients undergoing potentially curative treatment with liver transplantation, resection, or ablation, Sternby Eilard et al. evaluated non-liver comorbidities using a modified CCI that excluded liver disease. They demonstrated that greater comorbidity burden was associated with increased 5-year mortality even after adjustment for age and tumor burden, and several specific comorbid conditions, including cardiovascular, cerebrovascular, pulmonary, and renal diseases, were associated with mortality [26]. Similarly, Meischl et al. analyzed 1359 patients with HCC using a modified CCI that excluded both liver disease and HCC and found that increasing comorbidity burden was independently associated with poorer overall survival after adjustment for established prognostic factors, including liver function and tumor extent [27]. Notably, a high modified CCI was also associated with poorer liver-related survival in that study. In contrast, our analysis used a non-age-adjusted CCI, excluded HCC as the index disease, and retained other liver disease components included in the original CCI. These findings support the concept that comorbidity burden provides prognostic information beyond conventional tumor- and liver-related factors in HCC. Although previous studies included patients receiving various treatment modalities, our results extend this observation to patients treated with PBT and suggest that comorbidity assessment may also be relevant when estimating prognosis after definitive radiotherapy.

Recently, Lin et al. demonstrated that both the Charlson Comorbidity Index (CCI) and age-adjusted CCI (aCCI) were associated with survival outcomes in patients with HCC treated with transarterial chemoembolization combined with immune checkpoint inhibitors and targeted therapy [28]. Although the treatment modality and patient population differed from those in our study, both studies highlight the importance of patient-related factors in addition to tumor characteristics when estimating prognosis. Taken together, these findings suggest that assessment of comorbidity burden may improve risk stratification across different treatment modalities for HCC.

However, comorbidity burden represents only one component of the complex prognostic landscape of HCC. Tumor-related biological characteristics and the tumor microenvironment may also substantially influence disease progression and survival. Bi et al. analyzed gene-expression data from 342 patients with HCC and identified immune-evasion-associated genes within the tumor microenvironment, developing a six-gene prognostic risk model that was significantly associated with overall survival and achieved a C-index of 0.764 [29]. These findings illustrate that biological and immune-related characteristics may provide prognostic information beyond conventional clinical variables.

Metabolic and molecular mechanisms may also contribute to HCC behavior; Zheng et al. demonstrated through multi-omic analyses that modulation of amino acid metabolism and STAMBPL1-related signaling influenced malignant HCC phenotypes, further illustrating the biological complexity of HCC [30].

More recently, Ding et al. demonstrated the potential value of integrating multiple dimensions of information for individualized assessment of HCC. Using 1208 HCC lesions, they developed a multimodal model incorporating contrast-enhanced ultrasonography, magnetic resonance imaging, clinical variables, and biological information to predict tumor invasiveness and local tumor progression after thermal ablation. The model showed good discrimination in both internal and external test cohorts, and tumors classified as highly invasive exhibited distinct pathological and molecular features, including a higher frequency of microvascular invasion and upregulation of pathways related to angiogenesis, stress response, and tumor metastasis [31]. Taken together, these observations suggest that prognostic evaluation in HCC should ideally integrate patient-related factors such as comorbidity with liver function, tumor characteristics, imaging findings, and biological information rather than relying on any single clinical index. This study has several limitations. First, its retrospective single-center design may have introduced treatment-selection bias, because the study included only patients who were considered suitable for and ultimately received PBT. Comorbidities were also assessed retrospectively from medical records. In addition, local control was assessed according to routine clinical practice rather than a prospectively predefined uniform radiologic response criterion, which may have introduced variability in the assessment of local recurrence. Second, the CCI is a comorbidity-based index and does not capture all dimensions of patient condition or physiological reserve [32,33,34]. Frailty, nutritional status, sarcopenia, and other components of geriatric assessment were not systematically evaluated during the study period. Third, the cohort was treated between 2009 and 2015, and systemic therapy, imaging strategies, supportive care, and multidisciplinary management of HCC have evolved substantially since then; therefore, the applicability of these findings to contemporary practice should be interpreted with caution. Fourth, some age × CCI subgroups contained relatively small numbers of patients and events, limiting the statistical precision of subgroup comparisons (Table S1). In addition, formal competing-risk analysis was not performed, and post-recurrence treatments were individually selected according to tumor and patient characteristics and were not included in the present analysis. Finally, because this was a single-center Japanese cohort, regional differences in underlying liver disease, patient selection, and PBT practice may limit the generalizability of our findings.

## 5. Conclusions

In this cohort of patients with HCC treated with PBT, chronological age was not independently associated with OS or PFS, whereas a high comorbidity burden was independently associated with poorer OS; its association with PFS did not reach statistical significance. These findings suggest that comorbidity burden should be considered in addition to chronological age when estimating prognosis after PBT.

## Figures and Tables

**Table 1:** Backgrounds of elderly (≥75 years) and non-elderly (<75 years) patients.

Factors	Non-Elderly	Elderly	*p*-Value
N	164	105	
Age			
Median	66	80	
Range	39–74	75–96	
Gender (male)	135 (82.3)	74 (70.5)	0.034
Etiology			0.014
HBV	30 (18.3)	6 (5.7)	
HCV	76 (46.3)	58 (55.2)	
HBV + HCV	3 (1.8)	1 (1.0)	
Non-B/C	55 (33.5)	40 (38.1)	
Child-Pugh score			0.051
5	101 (61.6)	67 (63.8)	
6	27 (16.5)	28 (26.7)	
7	19 (11.6)	8 (7.6)	
8	7 (4.3)	1 (1.0)	
9	6 (3.7)	1 (1.0)	
10	4 (2.4)	0 (0.0)	
PLT (10^4^/µL) median (IQR)	11.75 (7.60–15.75)	13.20 (10.20–17.70)	0.028
AST median (IQR)	40.0 (28.0–57.0)	40.0 (29.0–55.0)	0.831
ALT median (IQR)	31.5 (20.0–47.5)	29.0 (20.0–47.0)	0.429
AFP median (IQR)	17.5 (5.97–216.25)	13.00 (5.00–79.00)	0.247
DCP median (IQR)	69.0 (24.0–552.0)	56.5 (20.0–479.5)	0.544
PVTT			0.008
Vp0–1	124 (75.6)	92 (88.5)	
Vp2	13 (7.9)	9 (8.7)	
Vp3	16 (9.8)	2 (1.9)	
Vp4	11 (6.7)	1 (1.0)	
Size (cm) median (IQR)	4.0 (2.5–6.5)	3.5 (2.4–5.1)	0.191
CCI			0.638
median (range)	1 (0–5)	1 (0–6)	
Total dose/fractions			0.422
66 Gy (RBE)/10 fr	26 (15.9)	21 (20.0)	
72.6 Gy (RBE)/22 fr	119 (72.6)	67 (63.8)	
74 Gy (RBE)/37 fr	17 (10.4)	16 (15.2)	
Others	2 (1.2)	1 (1.0)	
PS			0.011
0	91 (55.5)	47 (44.8)	
1	71 (43.3)	49 (46.7)	
2	2 (1.2)	6 (5.7)	
3	0	3 (2.9)	

PVTT data were unavailable for one elderly patient. Others included 70 Gy (RBE) in 35 fractions and 60 Gy (RBE) in 15 fractions.

**Table 2:** Multivariable Cox proportional hazards analyses for overall survival and progression-free survival.

Factors	OS HR (95% CI)	*p*-Value	PFS HR (95% CI)	*p*-Value
Age ≥ 75 vs. <75 years	1.246 (0.892–1.740)	0.198	1.154 (0.865–1.540)	0.331
CCI 1–2 vs. 0	1.337 (0.646–2.766)	0.434	1.162 (0.643–2.099)	0.620
CCI ≥ 3 vs. 0	3.313 (1.535–7.150)	0.002	1.873 (0.981–3.579)	0.057
Child-Pugh	2.422 (1.606–3.652)	<0.001	1.675 (1.143–2.454)	0.008
Tumor size	1.064 (1.006–1.126)	0.030	1.082 (1.031–1.135)	0.001
PVTT	1.452 (0.947–2.225)	0.087	1.515 (1.042–2.204)	0.030
AFP	1.364 (0.982–1.894)	0.064	1.123 (0.848–1.487)	0.417
PS	1.069 (0.775–1.475)	0.686	0.786 (0.592–1.044)	0.096
Sex	1.823 (1.247–2.664)	0.002	1.553 (1.108–2.176)	0.011
Etiology	1.034 (0.725–1.476)	0.852	0.914 (0.676–1.235)	0.557
Platelet count	1.004 (0.997–1.012)	0.288	1.002 (0.996–1.008)	0.616

**Table 3:** Treatment-Related Adverse Events.

Factors	Non-Elderly	Elderly
N	164	105
Acute (Grade 1–2)		
Dermatitis	44 (26.8)	45 (42.9)
Anorexia	0 (0.0)	1 (1.0)
Ascites	1 (0.6)	0 (0.0)
Acute (Grade ≥ 3)	0 (0.0)	0 (0.0)
Late (Grade 1–2)		
Pericardial effusion	0 (0.0)	1 (1.0)
Pleural effusion	2 (1.2)	2 (1.9)
Ascites	2 (1.2)	2 (1.9)
Telangiectasia	4 (2.4)	1 (1.0)
Rib fracture	7 (4.3)	1 (1.0)
Pneumonitis	2 (1.2)	0 (0.0)
Late (Grade ≥ 3)		
Gastrointestinal bleeding	1 (0.6)	0 (0.0)
Dermatitis	1 (0.6)	0 (0.0)

## Data Availability

The data that support the findings of this study are available from the corresponding author upon reasonable request. The data are not publicly available due to privacy and ethical restrictions.

## Supplementary Materials

The following supporting information can be downloaded at: https://www.mdpi.com/article/10.3390/cancers18183005/s1, Table S1: Number of patients and events according to age group and CCI category.

## Abbreviations

The following abbreviations are used in this manuscript: AFP Alpha-fetoprotein ALT Alanine aminotransferase AST Aspartate aminotransferase CCI Charlson Comorbidity Index CTCAE Common terminology criteria for adverse events DCP Des-γ-carboxy prothrombin HCC Hepatocellular carcinoma LC Local control OS Overall survival PBT Proton beam therapy PFS Progression-free survival PS Performance status PVTT Portal vein tumor thrombosis RBE Relative Biological Effectiveness.
