# Supplementary material for: Association of Chronological Age and Comorbidity Burden with Outcomes After Proton Beam Therapy for Hepatocellular Carcinoma
Source: Cancers (Basel). 2026 Sep 16;18(18):3005. doi: 10.3390/cancers18183005 (PMC13604634; doi:10.3390/cancers18183005)
Supplement: Supplementary file 1 [file cancers-18-03005-s001.zip › cancers-4528679-supplementary.pdf]

Supplementary Table S1. Number of patients and events according to age group and CCI category.

Age group	CCI	Patients, n	Deaths, n	PFS events, n
<75 years	0	9	3	7
<75 years	1–2	124	68	105
<75 years	≥3	31	23	26
≥75 years	0	8	6	7
≥75 years	1–2	81	58	69
≥75 years	≥3	16	13	15
